# Retraction: Outer Membrane Protein A (OmpA) of *Shigella flexneri* 2a Induces TLR2-Mediated Activation of B Cells: Involvement of Protein Tyrosine Kinase, ERK and NF-κB

**DOI:** 10.1371/journal.pone.0228299

**Published:** 2020-01-22

**Authors:** The PLOS ONE Editors

Following publication of this article [1], concerns were raised regarding Figure 2 and Figure 8:

- In Figure 2D/8A: when rotated 180°, lanes 1 and 2 of the α-tubulin panel of Figure 2D appear similar to the bands in lanes 3 and 4 of the Lamin B panel of figure 2D and lanes 4–5 of the Lamin B panel of Figure 8A.
- In Figure 8A, irregularities have been detected in the background of lanes 4–6 of the α-tubulin panel.
- In Figure 8A, at the right-hand side of lane 6 of the α-tubulin panel, there is a small area of signal with the appearance of a vertical edge.

The underlying western blot images for Figures 2 and 8 and the individual-level chart data for the other figures in the article are not available.

In light of the concerns about Figures 2D and 8A that question the integrity of the reported results, and the unavailability of the underlying data, the *PLOS ONE* Editors retract this article.

RB and DP agreed with retraction. MKC did not respond.
